# Impact of connected toothbrushes on patient perceptions of brushing skills and oral health: results from a randomized clinical trial and a single-arm intervention study

**DOI:** 10.1186/s12903-025-05907-5

**Published:** 2025-04-09

**Authors:** Juliana Gomez, LaTonya Kilpatrick, Maria Ryan, Chad Gwaltney

**Affiliations:** 1 Colgate-Palmolive Dental Health Unit, Manchester, UK; 2 Colgate-Palmolive Technology Center, Piscataway, NJ USA; 3 Gwaltney Consulting, Westerly, RI USA

**Keywords:** Patient-reported outcome, Qualitative interviews, Oral health behaviors, Connected toothbrush

## Abstract

Connected toothbrushes, where an electric toothbrush connects wirelessly to a smartphone app that provides feedback and guidance to the user, can improve clinical indicators of oral health. However, the impact of the connected brush on user perceptions of brushing skills, behaviors, “everyday” oral health (e.g., breath odor, how clean teeth feel), and user perceptions of the smartphone app are also important. In this study, a novel PRO measure – the Everyday Oral Health and Brushing (EOHAB) Questionnaire – was implemented to capture these important views. The EOHAB was administered in: (1) a randomized controlled clinical trial (RCT; *N* = 80) comparing a connected toothbrush to an unconnected electric brush over 6 weeks and (2) a single-arm study (*N* = 159), where participants used an electric brush alone for 1 week and then a connected brush for 3–4 weeks. EOHAB scores addressing perceptions of brushing behaviors/skills, oral health, and the usefulness and ease of use of the app were calculated. Impression of change items that directly asked users about changes in their perceptions were scored separately. In the single-arm study, perceptions of brushing skills and behaviors were very positive prior to using the unconnected or connected brush and then became incrementally more positive after using the electric brush alone and then the connected brush before decreasing at the last assessment interval. In both studies, cross-sectional correlations between the EOHAB scores and objective measures of brushing behavior and clinical measures were close to zero. Increased brushing duration and coverage over time were observed when participants reported improved perceptions on impression of change items and positive ratings of app utility, but not other EOHAB scores. In the RCT, the connected brush significantly improved several impression of change items, but not other EOHAB items asking about current perspectives. In sum, use of a connected toothbrush was associated with improved brushing and oral health perceptions when EOHAB questions asked directly about changes since starting use of the toothbrush. Brushing metrics improved as views on the app became more positive. Although not uniform, the results support previous studies indicating that connected brush systems can improve patient-reported outcomes.

## Background

Improving individual and population oral health is dependent upon improving routine at-home dental hygiene, including brushing habits. Advances in toothbrush technology can improve at-home brushing and clinical oral health outcomes: For example, electric toothbrushes are more effective at reducing plaque and gingivitis than manual toothbrushes in both adult and pediatric samples [1–5]. The development of novel “smart” or “connected” toothbrushes, enhance electric toothbrush technology through connectivity with mobile health (mHealth) applications. These connected toothbrushes provide users with personalized guidance, real-time feedback, and data-driven monitoring that are designed to improve brushing accuracy, duration, and adherence while also enabling clinicians to monitor patient progress remotely. With continued use, connected brushes may lead to improved oral health, plaque reduction through improved compliance, and the acquisition of new brushing skills and behaviors (e.g., the amount of time to brush, how to hold the toothbrush).

Evidence from multiple studies supports the superior efficacy of connected toothbrushes in improving oral health outcomes compared to manual or conventional electric toothbrushes. For instance, Goyal et al. [6] conducted a six-month randomized controlled trial comparing an oscillating-rotating smart toothbrush with a sonic toothbrush. Their findings showed that the connected toothbrush achieved significantly greater reductions in plaque and gingivitis, with sustained improvements throughout the study period. Similarly, Erbe et al. [7] demonstrated a 34% reduction in plaque among adolescents with orthodontic appliances using Bluetooth-enabled toothbrushes, compared to a minimal 1.7% reduction with manual brushes.

The potential of connected toothbrushes in enhancing periodontal health has also been highlighted. Tonetti et al. [8] emphasized their diagnostic capabilities, showing that self-reported bleeding on brushing, captured via connected toothbrushes, strongly correlated with clinical measures of periodontal inflammation. This ability to link real-time user data with clinical indicators underscores the role of connected brushes in monitoring periodontal health remotely. Similarly, Li et al. [9] demonstrated that AI-enabled multimodal toothbrushes, when combined with targeted mHealth messages, significantly reduced periodontal inflammation. In their randomized trial, users who received feedback from smart toothbrushes achieved a 7.9% greater reduction in periodontal inflammation compared to those using conventional methods. Together, these findings showcase the transformative potential of connected brushes in the prevention and management of periodontal diseases.

The effectiveness of app-integrated toothbrushes in reducing gingival bleeding has also been highlighted in recent research. Thurnay et al. [10] analyzed data collected from interactive toothbrushes and reported significant improvements in self-reported gingival bleeding scores. The study emphasized the value of personalized feedback in fostering healthier brushing habits and reducing soft tissue inflammation. Additionally, Erbe et al. [11] showed that adolescents using an interactive power toothbrush achieved superior plaque removal and increased brushing duration compared to those using manual brushes. The integration of connected toothbrushes with mHealth systems enables remote monitoring, allowing clinicians to access detailed brushing data and provide tailored recommendations. Flyborg [12] demonstrated the impact of this feature in a study where clinicians identified problematic brushing patterns through objective data analysis and intervened early, leading to significant improvements in oral health outcomes. In addition to changes in clinical outcomes, patient perceptions may also change following the use of a connected toothbrush. For example, a connected toothbrush may increase a patient’s confidence in their ability to brush effectively, their enjoyment of brushing, and their understanding of how to brush. Improvements in these perceptions are important outcomes in their own right and may serve as critical mediators of the effect of the novel brush on clinical outcomes. These changes in perspectives may allow for changes in brushing habits to persist when the connected toothbrush is not available or after it has been discontinued. Therefore, it is important to also measure these brushing perceptions in clinical trials and observational research.

Patient-reported outcome (PRO) measures include questions where respondents directly report on their experiences, perceptions and health status [13]. Several PRO measures have been used to measure various aspects of oral health, ranging from very narrow and specific assessments of pain to broad assessments of oral health-related quality of life [14–16; e.g., Oral Health Impact Profile, General Oral Health Assessment Index]. The Everyday Oral Health and Brushing (EOHAB) Questionnaire was developed as a PRO measure to examine perspectives on “everyday” oral health and dental hygiene behaviors and perceptions that may be important mediators of subsequent clinical outcomes, and views on “connected” electric toothbrush systems. The EOHAB includes items that ask about the “past week” and items that directly ask about changes that the respondent has experienced since starting to use the connected toothbrush. The latter “impression of change” items may be valuable in studies evaluating brushing perceptions: many people may believe that they have excellent brushing skills and oral health, which makes it difficult to demonstrate connected toothbrush benefits on PRO items that ask respondents to rate their brushing skills and behaviors at that time (ceiling effects).

The EOHAB was used to evaluate the impact of a connected toothbrush on user perceptions of oral health and brushing skills and behaviors. EOHAB data were collected in 2 studies: (1) a randomized controlled clinical trial comparing a connected toothbrush to an unconnected electric brush and (2) a single-arm study, where participants first used an electric brush alone and then used a connected brush. Clinical outcomes from the randomized controlled trial have been reported elsewhere [17].

## Methods

EOHAB data were collected from participants (1) in a randomized controlled trial (RCT) evaluating the effect of using a connected toothbrush on plaque and gingivitis (ClinicalTrials.gov Identifier: NCT04221334, registration date 30 September 2019) and (2) a single-arm study evaluating longitudinal use of a connected toothbrush.

### Participants

All participants provided informed consent before participating in the study.

#### RCT sample

Participants in the RCT were recruited through the clinical site (Loma Linda University, Loma Linda, CA). Participants were required to (a) be between 18 and 70 years old, (b) be available for the 6 weeks study duration, (c) have a minimum of 20 natural uncrowned teeth (excluding 3rd molars) present, (d) have initial mean gingival index of at least 1.0 as determined by Loe and Silness Gingival Index, (e) have an initial mean plaque index of at least 0.6 as determined by Rustogi Modification of the Navy Plaque Index, (f) be in good general health, (g) have no known history of allergy to personal care/consumer products or their ingredients, relevant to any ingredients in the test products as determined by the study examiner, and (h) be fluent in English. Participants could not have any contraindicated medical or dental conditions or medications.

#### Single-arm study sample

Participants in the single-arm study were recruited through a clinical site (Eurofins CRL, Piscataway, NJ). Participants were required to (a) be between 18 and 70 years old, (b) be available for the 5-week study duration, (c) be in good general health, (d) have access to a smartphone or tablet, (e) be willing to download and use the app associated with the connected brush and discontinue use of personal toothbrush during the study, (f) speak fluent English, (g) be willing to inform the investigating site of any dental cleanings or procedures that took place over the course of the study, and (h) be willing to continue using regular toothpaste, flossers, and mouthwash, as applicable for the duration of the study. Participants could not have any contraindicated medical or dental conditions or medications.

### Measures

#### EOHAB

Two versions of the EOHAB were used in the RCT and single-arm study. The RCT was conducted first and included an initial draft of the EOHAB. The initial draft EOHAB was based on qualitative pilot interviews with 7 individuals who had used the connected brush for 4 weeks. This version included 11 items assessing brushing behaviors (brushing frequency), perceptions of brushing effects (how clean teeth felt), perspectives on brushing (how enjoyable to brush, effectiveness of brushing, motivation to brush), and brushing skills (confidence in brushing skills, focus on brushing when brushing) in the past 7 days. Five items asked participants directly about changes in their brushing behaviors and skills since they started using the study toothbrush. The EOHAB was further updated following qualitative interviews with 10 participants in the RCT. These interviews addressed participant comprehension of the EOHAB instructions, items, and response scales and the relevance of the measure’s content. The subsequent EOHAB version used in the single-arm study included 39 items, with 17 items assessing oral health and brushing experiences, behaviors, and outcomes over the past 7 days (Item Set 1), 7 items measuring changes in brushing since starting use of the study toothbrush (Item Set 2), and 15 items assessing the usability and usefulness of the connected toothbrush app over the last 7 days (Item Set 3). Response scales across the 2 versions included a mix of 0–10 numeric rating scales, 5-point verbal response and Likert-type scales, and dichotomous true/false statements.

#### Rustogi modification of navy plaque index [RM-NPI; 18]

The RM-NPI was used to evaluate dental plaque in the RCT only. The total plaque scores range from 0.0 to 1.0, with higher values indicating the presence of plaque in more areas.

#### Löe-silness gingival index [LSGI; e.g., 19]

The LSGI was used to evaluate gingivitis severity. The total mouth score was categorized using the following rules: from 0.1 to 1.0 = Mild inflammation - slight change in color, slight edema, no bleeding on probing; 1.1-2.0 = Moderate inflammation - redness, edema, glazing, bleeding on probing, and 2.1-3.0 = Severe inflammation - marked redness and edema, ulceration, tendency toward spontaneous bleeding [e.g., 20].

#### Objective brushing metrics

The electric toothbrushes used in the studies were capable of collecting objective data on brushing, including the following:

- Time and date stamps were assigned to each brushing episode, allowing for frequency of brushing to be calculated.
- Duration of each brushing episode (in seconds).
- Portion of the whole mouth that received coverage/brushing during each brushing episode (as a percentage).

### Procedures

The RCT was a randomized, single-center, two-group, examiner-blind and parallel-group study comparing a connected power toothbrush and smartphone app (hum by Colgate) to a non-connected power toothbrush (*N* = 80). The non-connected brush was equivalent to the connected brush, but without Bluetooth capability. Therefore, the non-connected brush could not connect to the app. Assessments were completed at Baseline, and 3- and 6-weeks after randomization. Changes in dental plaque and gingivitis over a 6-week period were the primary and secondary endpoints of the RCT, respectively. The effects of the connected toothbrush on the primary and secondary endpoints are reported elsewhere [17]. The sample size was chosen to have 80% power to detect a between-group difference of 0.04 units on the primary endpoint at the 0.05 significance level. RM-NPI and LSGI were completed only in the RCT and were administered at Screening and at both pre-brushing and post-brushing examinations at the Baseline, 3-weeks, and 6-week clinic visits. The EOHAB items were administered at Baseline, and the 3- and 6-week follow-up intervals. Objective brushing metrics (duration, coverage, frequency) were only collected from the participants in the connected toothbrush condition.

The single-arm study involved 5 weeks of electric toothbrush use (*N* = 159): 1 week using the electric toothbrush alone and 4 weeks using the connected toothbrush and smartphone app (hum by Colgate). The sample size was chosen to have a sufficient number of participants to conduct correlational analyses and factor analysis of the measure’s internal structure. Assessments were completed at baseline prior to initiating use of the non-connected electric toothbrush (Baseline 1), after 1 week of non-connected electric toothbrush use (Baseline 2), after 1 week of connected toothbrush use (Post-Baseline 1), and after 3 additional weeks of connected toothbrush use (Post-Baseline 2). EOHAB items asking about perspectives on brushing and oral health in the past 7 days were completed at all assessment intervals. EOHAB items asking about changes since using the study brush were asked at all intervals, except Baseline 1. EOHAB items asking about perspectives on the smartphone app were completed at the Post-Baseline 1 and 2 intervals. Objective brushing metrics were available at all intervals except Baseline 1.

### Data management and analysis

For the objective brushing variables collected by the hum by Colgate toothbrush, weekly brushing coverage and duration averages were calculated across all brushing instances from the seven days prior to a target timepoint, while a weekly sum was used for the number of brushing episodes.

Data were initially examined by frequency tables and item intercorrelation matrices. During this process, it was found that two of the items related to the EOHAB items asking about the smartphone app had duplicate responses (“How much did you learn from the app about the areas in your mouth that you need to brush?” and “How much did you change the way that you brush your teeth due to the app?”). This was likely due to an error in data coding, and, therefore, only one of the variables was retained for use in analysis (the item asking about change in brushing behavior).

Data analyses were designed to evaluate the optimal method for scoring the EOHAB items, relationships between the EOHAB items and objective plaque, gingivitis, and brushing metrics, and longitudinal changes in EOHAB scores relative to type of toothbrush being used.

- *EOHAB Scoring.* Exploratory and confirmatory factor analysis (EFA and CFA, respectively) models were used to identify a scoring algorithm for the EOHAB Item Sets 1 and 3 separately. Item Set 2 was not included, as the global impression of change items were designed to be analyzed independently and not as an aggregate score. EFA and CFA of Item Set 1 used Baseline 1 data from the observational study and analysis of Item Set 3 used the Post-Baseline 1 values. Both sets of analyses were completed in MPLUS 8.4 [21] using robust maximum likelihood estimation when item responses were treated as continuous or weighted least squares estimation with mean and variance correction (WLSMV) when item responses were treated as categorical. Model fit of the CFA models were examined against the Confirmatory Fit Index [CFI; 22], the Tucker-Lewis index [TLI; 23], and the root mean square error of approximation [RMSEA; 24], using customary cut-offs for adequate fit of 0.95 or greater for the TLI and CFI [25] and less than 0.08 for the RMSEA [26]. Cronbach’s alpha, a measure of internal consistency reliability or how strongly the items of a measure are related to each other, was calculated for the derived factors (range: 0–1, with higher scores indicating better reliability).
- *Relationships Between EOHAB and Other Variables.* Descriptive analyses and unconditional and conditional longitudinal methods were used to examine changes in EOHAB scores over time in the single-arm study. A series of random effects models [e.g., 27] were fitted within the Item Set 1 and Item Set 3 data. Pearson correlations were calculated to evaluate the relationships between the EOHAB scores and the objective brushing metrics and clinical variables both cross-sectionally and longitudinally.
- *Connected Brush Effects.* Effects of the connected brush on EOHAB scores were evaluated from the RCT data. Change scores from Baseline to Week 3 and Baseline to Week 6 were calculated and the treatment groups were compared using independent groups t-tests for continuous variables and logistic/ordinal regression for binary or Likert-type scales. Cohen’s d values were calculated for the t-tests to provide a standardized measure of treatment effect size across continuous outcomes [28] and odds ratios are presented for the regression analyses to depict the magnitude of the treatment differences.

## Results

### Participant characteristics

Descriptive statistics for all demographic variables are reported in Table 1. Table 1 also includes baseline scores for the plaque and gingivitis measures in the RCT.

**Table 1:** RCT and Single-Arm study participant characteristics

Characteristic	Single-Arm Study (*N* = 159)	RCT (*N* = 80)
Age	43.4 (14.8)	37.6 (12.6)
Gender	76.7% Female 23.3% Male	63.8% Female 36.2% Male
Race/Ethnicity	10.7% Asian 28.3% Black or African-American 10.7% Hispanic 10.1% Indian 1% Middle Eastern 5% Pakistani 1% Pacific Rim 33% White 1% Other	8.8% Asian 7.5% Black or African-American 42.5% Hispanic 37.5% White 3.8% Other
RM-NPI Total Mouth Score	Not collected	0.7 (0.1)
LSGI Total Mouth Score	Not collected	1.5 (0.2)

RM-NPI *=* Rustogi Modification of Navy Plaque Index; LSGI = Löe-Silness Gingival Index

### Item-level descriptive statistics

Descriptive statistics for Item Sets 1 and 3 from Baseline 1 and 1-week post-connected brush use, respectively, in the single-arm study are provided in Table 2. Item-level summaries were examined for floor effect, ceiling effect, and missing data to identify items that may be performing sub-optimally (i.e., responses largely grouped in the least positive [floor effect] or most positive response category [ceiling effect]). All items that used a 0–10 response scale had few responses in the lower portion of the response scale (i.e., response options 0–4); the vast majority of responses were between 5 and 10. For Item Set 1, this indicates that participants already had very positive views on their brushing and oral health *prior to* using the non-connected or connected toothbrush in the single-arm study. For Item Set 3, the score distributions indicate that participants had very positive views on the use of the app following the first week of connected brush use in the single-arm study.

**Table 2:** Descriptive statistics for EOHAB item set 1 (Baseline 1; first assessment interval) and item set 3 (Post-Baseline 1; 1-Week after connected brush Use) in Single-Arm study

EOHAB Item (Response Scale Range)	Mean	SD
Item Set 1		
*In the past 7 days…*		
Brushed teeth how often (0–4)^a^	2.01	0.58
Breath smell (0–4)^b^	2.82	0.69
Teeth felt clean after brushing (0–10)	7.57	1.87
Teeth felt clean throughout day (0–10)	6.69	2.03
Teeth looked clean after brushing (0–10)	7.53	1.98
Teeth looked clean throughout day (0–10)	6.85	2.14
Easy to brush teeth (0–10)	8.44	1.88
Enjoyable to brush teeth (0–10)	7.11	2.31
Teeth brushed how well (0–10)	7.33	2.06
Satisfaction with teeth brushing effectiveness (0–10)	7.06	1.97
Motivation for brushing teeth (0–10)	7.50	2.31
Confidence in brushing teeth correctly (0–10)	6.95	2.34
Focused on brushing while brushing teeth (0–10)	6.92	2.51
Attentive about brushing time while brushing teeth (0–10)	6.62	2.74
Satisfaction with toothbrush (0–10)	6.56	2.45
Bleeding from gums during brushing (0–10)	7.92	2.97
Confidence about teeth brushing effectiveness (0–4)^c^	2.78	0.82
Item Set 2		
App was helpful when brushing teeth (0–10)	8.85	2.05
App helped for brushing right length of time (0–10)	9.21	1.75
App helped for brushing in right locations (0–10)	9.18	1.56
Learned from app about brushing right length of time (0–10)	9.22	1.76
App changed how you brushed teeth (0–10)	9.03	1.78
Feedback from app was helpful (past 7 days; 0–10)	8.89	2.03
Weekly summaries were helpful (0–10)	8.60	2.07
Daily summaries were helpful (past 7 days; 0–10)	9.25	1.40
App was easy to use (past 7 days; 0–10)	9.39	1.29
Easy to see app-generated mouth image (past 7 days; 0–10)	8.97	1.65
App accuracy tracking brushing time (past 7 days; 0–10)	8.48	2.13
App accuracy tracking brushing location (past 7 days; 0–10)	9.41	1.41
Easy to connect toothbrush to app (past 7 days; 0–10)	8.71	2.06
Use of toothbrush without app (past 7 days; 0–4)^d^	2.71	0.85

All items rescaled, so that higher scores indicate more positive perspectives on brushing, oral health, and app use

^a^ 0 = Less than once a day, 1 = Once a day, 2 = Twice a day, 3 = Three times a day, 4 = More than 3 times a day. ^b^ 0 = Very bad, 1 = Bad, 2 = Neither good nor bad, 3 = Good, 4 = Very good. ^c^ 0 = Strongly disagree, 1 = Disagree, 2 = Neither agree nor disagree, 3 = Agree, 4 = Agree. ^d^ 0 = Always, 1 = Often, 2 = Sometimes, 3 = Rarely, 4 = Never. 0 = Worst possible response, 10 = Best possible response on 11-point response scales

SD = Standard Deviation

### Scoring algorithms

EFA and CFA models were examined for Item Set 1 using data from Baseline 1 in the single-arm study. The scree plot of eigenvalues suggested a 2-factor solution, so EFAs were conducted for both 2 and 3 factor solutions using oblique Crawford Ferguson-quartimax rotation to rotate initial factor extraction values to more readily interpretable solutions.

An a priori unidimensional confirmatory model was fit to Item Set 1 items (Table 3). This model provided a poor fit to the data, with all three model fit indices failing to meet their stated cut-thresholds for acceptable fit. Additionally, the factor loadings for item 1 (“Brushed teeth how often”) and item 16 (“Bleeding from gums during brushing”) were extremely low suggesting that these items should be considered separately from the rest of the Item Set 1 items. These items were dropped from subsequent CFA models. A two-dimensional model in which items related to teeth/mouth feel versus brushing experience loaded onto separate factors improved the model fit (CFI and TLI are above their threshold to define acceptable fit) but the RMSEA (observed value = 0.13) did not meet the acceptable fit criterion (≤ 0.08). A bifactor model in which all items load on a “general” factor and on no more than one “specific” factor was developed. Based on the factor loadings of the two-dimensional solution, the decision was also made to remove items 2 (Breath smell) and 17 (Confidence in teeth brushing effectiveness) which had the lowest loadings on their respective factors. The final bifactor model, fit to the remaining 13 items had the best fit of the examined models (RMSEA = 0.10, CFI = 0.99; TLI = 0.98) as two of the three fit indices were at acceptable levels and the RMSEA was close to the acceptable fit cut-value. The general and specific factor 2 dimensions were retained (Table 3). The general score is named as an Overall Summary Score and the score corresponding to factor 2 is a Brushing Experience score. The additional factor, which includes items addressing the look and feel of the teeth and how easy it is to brush teeth, is not analyzed independently (the items are included in the Overall Summary Score). Cronbach’s alpha was 0.95 for the Overall Summary Score and 0.91 for the Brushing Experience score. Both values indicate excellent internal consistency reliability.

**Table 3:** CFA results for item set 1 from baseline 1 (first assessment) in Single-Arm study

Item #	Item content	1 Factor	2 Factor		Bifactor		
		F1	F1	F2	Gen	S1	S2
1	Brushed teeth how often	0.13					
2	Breath smell	0.45	0.50				
3	Teeth felt clean after brushing	0.77	0.82		0.72	0.38	
4	Teeth felt clean throughout day	0.84	0.88		0.76	0.42	
5	Teeth looked clean after brushing	0.83	0.88		0.80	0.32	
6	Teeth looked clean throughout day	0.89	0.93		0.77	0.64	
7	Easy to brush teeth	0.73	0.79		0.76	0.12	
8	Enjoyable to brush teeth	0.74		0.76	0.68		0.34
9	Teeth brushed how well	0.84		0.86	0.89		0.02
10	Satisfaction with teeth brushing effectiveness	0.86		0.89	0.90		0.10
11	Motivation for brushing teeth	0.78		0.80	0.69		0.41
12	Confidence in brushing teeth correctly	0.88		0.89	0.78		0.45
13	Focused on brushing while brushing teeth	0.93		0.94	0.81		0.50
14	Attentive about brushing time while brushing teeth	0.84		0.85	0.66		0.63
15	Satisfaction with toothbrush	0.86		0.88	0.79		0.39
16	Bleeding from gums during brushing	0.14					
17	Confidence about teeth brushing effectiveness	0.67		0.68			
Model Fit							
	RMSEA	0.16	0.13		0.10		
	CFI	0.94	0.97		0.99		
	TLI	0.93	0.96		0.98		

F1 = Factor 1, F2 = Factor 2, Gen = Overall Score, S1 = Look and Feel of Teeth/Ease of Brushing, S2 = Brushing Experience. Items 1, 2, 16, and 17 were not included in the final factor solution and should be analyzed separately from the aggregate scores. Model fit indices: RMSEA = root mean square error of approximation (adequate fit < 0.08); CFI = Confirmatory Fit Index (adequate fit ≥ 0.95); TLI = Tucker-Lewis index (adequate fit ≥ 0.95)

For Item Set 3, the scree plot and examination of the eigenvalues suggested that 2 factors were likely appropriate for extraction in EFAs. Three models were examined in the CFA: an a priori unidimensional model, a two-factor model based on the two-factor EFA solution and a bifactor model, in which all items loaded onto a general factor and 1 of 2 specific sub-domain factors. The factor loadings and overall model fit of these models are provided in Table 4. Item app15 (“Use of toothbrush without app”) displayed a factor loading value noticeably lower than all other items; based on this finding, app15 was removed from further dimensionality analyses. While the correlated two-factor model was supported by the strong loadings for all remaining items and the observed CFI and TLI values, the RMSEA was well above the previously noted cut-off threshold for acceptable fit. The bifactor model fit well (RMSEA = 0.08; CFI = 1.00; TLI = 0.99), had factor loading values that were sufficiently large on the general factor (0.50 to 0.95), and the assignment of items onto the specific factors were substantively interpretable (i.e., Factor 1 as an “App Utility” factor and Factor 2 as an “App Usability” factor). From these analyses, the preferred structure of the items was set as the bifactor model. All three scores had coefficient alpha estimates above 0.80 (0.93, 0.94, and 0.81, respectively), which is above the stated minimum threshold of 0.70 for acceptable internal consistency reliability.

**Table 4:** CFA results for item set 3 from Post-Baseline 1 (1-Week after connected brush Use) in Single-Arm study

POCT-Q Item ID		1 Factor	2 Factor		Bifactor		
	Item content	F1	F1	F2	Gen	S1	S2
app1	App was helpful when brushing teeth	0.88	0.89		0.90	-0.12	
app2	App helped for brushing right length of time	0.92	0.93		0.90	-0.33	
app3	App helped for brushing in right locations	0.91	0.92		0.88	-0.34	
app4	Learned from app about brushing right length of time	0.84	0.86		0.82	-0.35	
app5	Learned from app about brushing right areas						
app6	App changed how you brushed teeth	0.68	0.70		0.68	-0.30	
app7	Feedback from app was helpful (past 7 days)	0.88	0.90		0.92	0.01	
app8	Weekly summaries were helpful	0.92	0.96		0.93	0.25	
app9	Daily summaries were helpful (past 7 days)	0.91	0.98		0.95	0.29	
app10	App was easy to use (past 7 days)	0.84		0.82	0.60		0.66
app11	Easy to see app-generated mouth image (past 7 days)	0.68		0.89	0.72		0.45
app12	App accuracy tracking brushing time (past 7 days)	0.67		0.76	0.64		0.38
app13	App accuracy tracking brushing location (past 7 days)	0.76		0.88	0.76		0.20
app14	Easy to connect toothbrush to app (past 7 days)	0.73		0.78	0.50		0.80
app15	Use of toothbrush without app (past 7 days)	0.31					
Model fit							
	RMSEA	0.15	0.13		0.08		
	CFI	0.97	0.99		1.00		
	TLI	0.97	0.98		0.99		

F1 = Factor 1, F2 = Factor 2, Gen = Overall Score, S1 = App Utility, S2 = App Usability. Item 15 was not included in the final factor solution and should be analyzed separately from the aggregate scores. Model fit indices: RMSEA = root mean square error of approximation (adequate fit < 0.08); CFI = Confirmatory Fit Index (adequate fit ≥ 0.95); TLI = Tucker-Lewis index (adequate fit ≥ 0.95)

### Longitudinal descriptive statistics

**Fig. 1:**
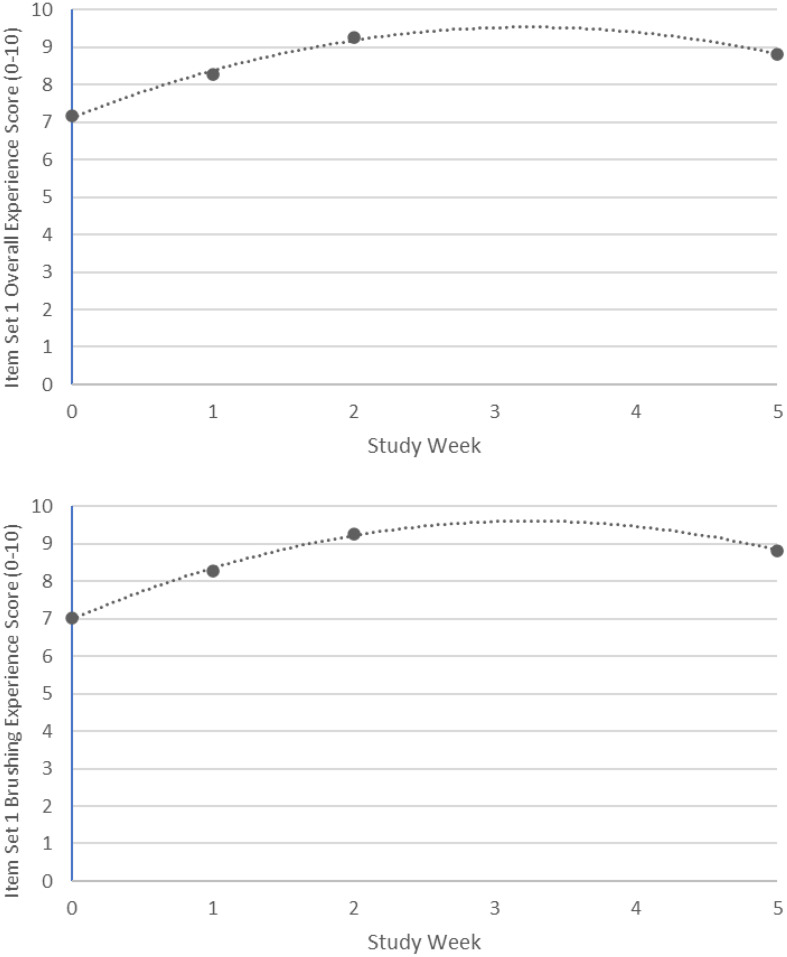
Changes in EOHAB Item Set 1 Overall Experience and Brushing Experience Scores Over Time in Single-Arm Study. Week 0 = Assessment before unconnected brush use; Week 1 = Assessment before connected brush use; Week 2 = Assessment 1 week after connected brush use; Week 5 = Assessment after 4 weeks of connected brush use

Figure 1 displays changes over time in the Item Set 1 scores from the single-arm study. Unconditional growth curve models were fit to each of the available EOHAB Item Set 1 scores to determine the optimal functional form for describing change over time. A quadratic time trend provided the best fit to the data. To better understand this trend, instantaneous rates of change (IROCs; how much scores are changing at a particular point in time) were estimated from the quadratic time trend model. From Baseline 1 through Week 1 post BL2, scores were significantly increasing over time but the IROCs overall scores became less positive (Overall Summary Score: BL1 IROC = 1.50, *p* <.0001; BL2 IROC = 1.03, *p* <.0001; Week1 Post BL2 IROC = 0.57, *p* <.0001; Brushing Experience: BL1 IROC = 1.63, *p* <.0001; BL2 IROC = 1.13, *p* <.0001; Week1 Post BL2 IROC = 0.62, *p* <.0001). The trajectory of the scores peaks after 1 week of using the connected brush and decreases through the end of the study (Overall Summary Score: Week 4 Post BL2 IROC=-0.82, *p* <.0001; Brushing Experience: Week 4 Post BL2 IROC=-0.89, *p* <.0001).

**Fig. 2:**
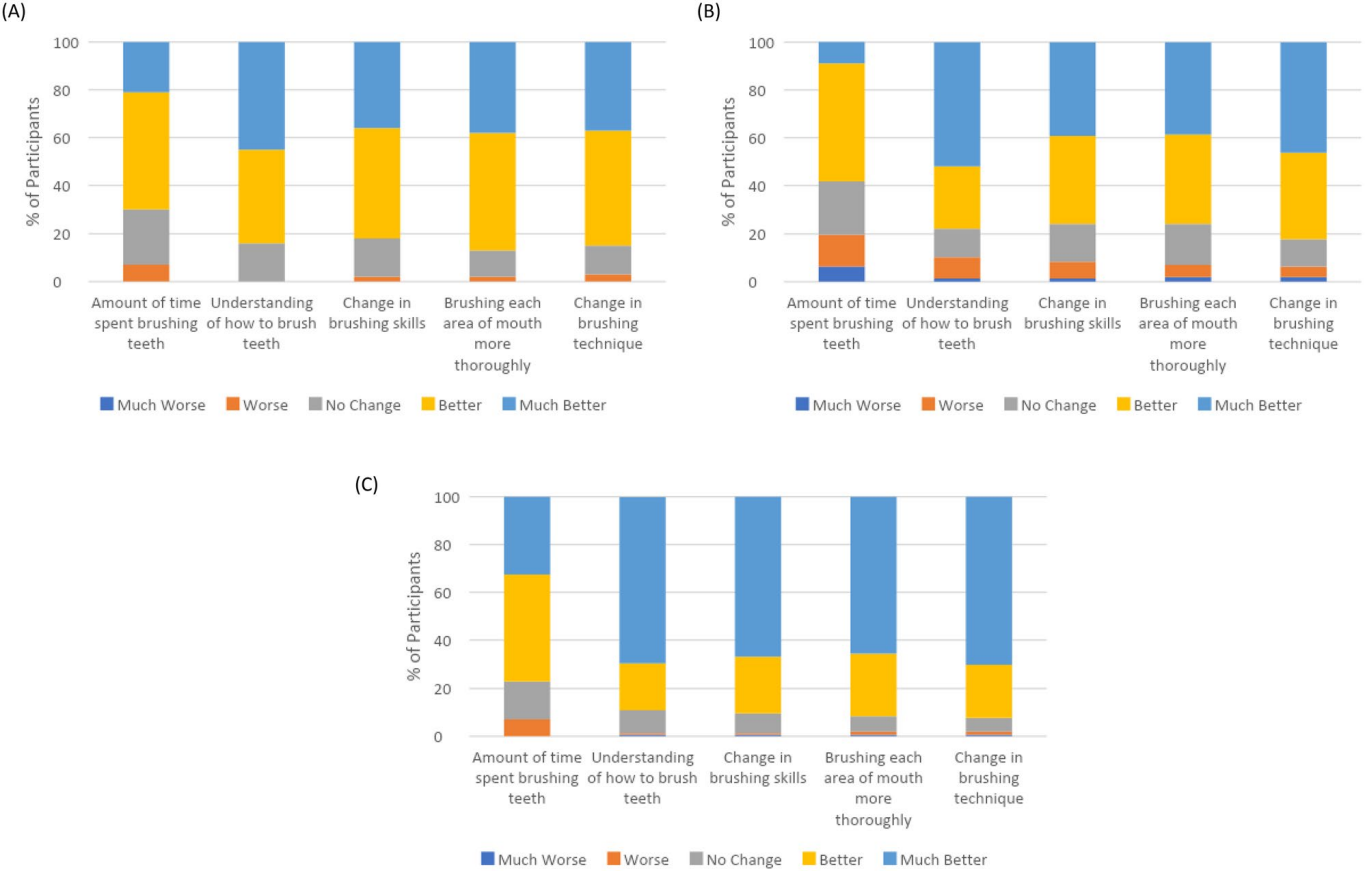
Item Set 2 Change Items Using 5-Point Response Scale in Single-Arm Study (**A**) 1 Week After Non-Connected Brush Use No participants responded “Much Worse” to the items. (**B**) 1 Week After Connected Brush Use (**C**) 4 Weeks After Connected Brush Use No participants responded “Much Worse” to the items

Item Set 2 items inherently ask about change over time in brushing behaviors and perspectives. The values for the individual items using a 5-point response scale (Much Worse to Much Better) at Baseline 2 and 1 and 3 weeks after starting to use the connected brush in the single-arm study are included in Fig. 2. The values indicate that participants generally perceived improvements in their brushing behaviors and skills throughout the single-arm study. After 3 weeks of connected brush use, approximately 80% or more of participants perceived improvements in brushing, with higher percentages of participants reporting improvements on items assessing understanding of how to brush teeth, change in brushing skills, brushing each area of the mouth more thoroughly, and change in brushing technique. The percentage reporting an improvement was slightly less for the item asking about amount of time spent brushing teeth. Two additional change items asked about changes and improvements in “the way that I brush” using a binary Yes/No scale. At Baseline 2, after using the electric brush alone in the single-arm study, 37% of participants responded “Yes” to each of the items. However, at 1-week after using the connected brush, these values increased substantially: 90% indicated that their brushing had changed and 87% indicated that it had improved. At 3-weeks, these values were 92% and 95%, respectively.

**Fig. 3:**
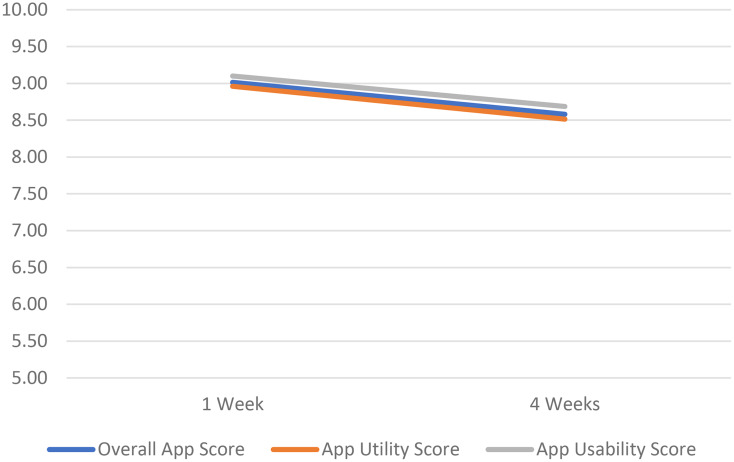
Item Set 3 Scores In Single-Arm Study Week 1 *n* = 158, Week 3 *n* = 157 1 Week = 1 week after initiation of connected brush use; 4 Weeks = 4 weeks after initiation of connected brush use; All scores are on 0–10 response scale

Figure 3 displays the changes in the average Item Set 3 scores at 1 and 4 weeks after starting to use the connected brush in the single-arm study. The scores range from 0 to 10, with higher scores indicating more positive views of the app. Scores were high (> 8.5) at both time points indicating a favorable view on app usefulness and usability, but the scores decreased from Week 1 to Week 4.

### Relationships with clinical and objective brushing variables

#### Cross-sectional correlations

Correlations between the Item Sets 1 and 3 summary scores and the clinical and objective brushing reference variables (brushing duration, brushing frequency, and brushing coverage) are included in Table 5. For Item Set 1, correlations were near zero at Baseline 1 and cross-sectional correlations at other timepoints were similar in magnitude. The cross-sectional correlations between the Item Set 1 scores (based on the available items) and the clinical variables in the RCT at Baseline were also near 0. For Item Set 3, the cross-sectional correlations at 1-week post-baseline in the single-arm study were also near 0.

**Table 5:** Cross-Sectional pearson correlations between EOHAB scores and clinical and objective brushing scores in Single-Arm study and RCT

EOHAB Score	Single-Arm Study			RCT	
	Brushing Frequency	Brushing Duration	Brushing Coverage	RM-NPI	LSGI
Item Set 1	Baseline 1 (First Assessment)			Baseline	
Overall Summary Score	-0.05	0.01	0.02	-0.05	-0.02
Brushing Experience	-0.03	0.05	0.07	-0.05	-0.02
Item Set 3	After 1 Week of Connected Brush Use				
Overall App Score	0.07	-0.03	-0.08	-	-
App Utility Score	0.03	-0.02	-0.07	-	-
App Usability Score	0.16	-0.02	-0.07	-	-

Item Set 3 data were not collected in the RCT

#### Sensitivity to change

Correlations between changes over time on the Item Sets 1, 2, and 3 summary scores with changes on the clinical and objective brushing reference variables are included in Table 6. Correlations were near zero for Item Set 1 scores in both the single-arm study and RCT. Item Set 2 global impression of change scores were not correlated with brushing frequency or the clinical variables, but several items were significantly correlated with brushing duration and coverage; the correlations indicate that participant reports of improved brushing skills and behaviors were associated with increased brushing duration and coverage. For Item Set 3, the Overall App Score and App Utility Score were significantly correlated with brushing duration and coverage, indicating that duration and coverage increased with increased positive perspectives about the app. The App Usability Score was not related to any brushing metric.

**Table 6:** Pearson correlations between changes in EOHAB scores and clinical scores in RCT and objective brushing scores in Single-Arm study

EOHAB Score	Single-Arm Study (Before Beginning Connected Brush to 4 Weeks After Connected Brush Use)			RCT (Baseline to 6 Weeks Post-Randomization)	
	Brushing Frequency	Brushing Duration	Brushing Coverage	RM-NPI	LSGI
*Item Set 1*					
Overall Summary Score	-0.02	0.09	0.10	-0.02	-0.02
Brushing Experience	-0.05	0.08	0.12	-0.03	0.01
*Item Set 2*					
Amount of time spent brushing teeth	0.04	0.22*	0.13	0.00	-0.13
Understanding of how to brush teeth	0.01	0.34***	0.22*	-0.01	0.01
Change in brushing skills	0.09	0.28**	0.16	-	-
Brushing each area of mouth more thoroughly	-0.07	0.29**	0.18*	-0.02	-0.15
Change in brushing technique	0.02	0.27**	0.17	-0.03	-0.07
Changed the way I brush	-0.01	0.29**	0.19*	-	-
Improved the way I brush	0.04	0.45***	0.28**	-0.12	-0.03
*Item Set 3*					
Overall App Score	0.07	0.24**	0.25**	-	-
App Utility Score	0.07	0.29***	0.27**	-	-
App Usability Score	0.04	0.09	0.14	-	-

*p <.05; **p <.01; ***p <.001

Item Set 3 was not administered in the RCT

### Effects of connected brush on EOHAB scores: RCT

The effects of connected toothbrush use on the EOHAB Item Sets 1 and 2 in the RCT are described in Table 7. The connected brush did not improve the Overall Summary Score or Brushing Experience Score from Item Set 1 relative to the unconnected brush. At Weeks 3 and 6, the scores in both groups were increased reflecting improved perspectives on brushing, but the improvement was modest in magnitude (< 1 point on the 0–10 scale). In contrast, all but 1 of the Item Set 2 impression of change scores were significantly greater in the connected brush group at Week 3; more participants reported an improvement in the amount of time spent brushing their teeth, a greater understanding of how to brush their teeth, brushing each area of the mouth more thoroughly, and an improvement in their brushing technique. At Week 6, participants in the connected brush group reported that they increased the amount of time that they spent brushing their teeth more than the non-connected group, but none of the other comparisons were statistically significant.

**Table 7:** Effects of connected brush on item sets 1 and 2 in RCT at weeks 3 and 6 Post-Randomization

EOHAB Score	Connected Brush	Non-Connected Brush	Treatment Effect
**Item Set 1**	**Change from Baseline**		
**Week 3**			
Overall Summary Score	0.44 (2.41)	0.51 (2.05)	d = -0.03
Brushing Experience	0.54 (2.65)	0.33 (2.30)	d = 0.08
**Week 6**			
Overall Summary Score	0.35 (2.26)	0.41 (2.56)	d = -0.03
Brushing Experience	0.32 (2.44)	0.34 (2.69)	d = -0.01
**Item Set 2**	**Change Rating**		
**Week 3**			
Amount of time spent brushing teeth	3.03 (0.92)	2.43 (0.71)	OR = 4.90, *p* <.001
Understanding of how to brush teeth	3.25 (0.93)	2.65 (0.86)	OR = 3.68, *p* <.01
Brushing each area of mouth more thoroughly	3.23 (0.97)	2.88 (0.72)	OR = 2.92, *p* <.05
Change in brushing technique	3.20 (0.91)	2.63 (0.81)	OR = 4.20, *p* <.01
**Week 3**	**Percent of Respondents**		
Improved the way I brush	85% True	80% True	OR = 1.42, ns
**Week 6**	**Change Rating**		
Amount of time spent brushing teeth	2.95 (1.04)	2.58 (0.78)	OR = 2.63, *p* <.05
Understanding of how to brush teeth	3.18 (0.96)	3.03 (0.86)	OR = 1.50, ns
Brushing each area of mouth more thoroughly	3.10 (1.03)	2.88 (0.85)	OR = 1.89, ns
Change in brushing technique	3.30 (1.02)	3.05 (0.88)	OR = 2.08, ns
**Week 6**	**Percent of Respondents**		
Improved the way I brush	87.5% True	80% True	OR = 1.75, ns

d = Cohen’s d (effect size); OR = Odds Ratio; Amount of time spent brushing: 1 = A lot shorter/A little shorter, 2 = Neither shorter nor longer (no change), 3 = A little longer, 4 = A lot longer; Understanding of how to brush teeth: 1 = Much worse/Little worse, 2 = Unchanged (the same), 3 = Little better, 4 = Much better; Brushing each area of the mouth more thoroughly: 1 = Strongly disagree/Disagree, 2 = Neither agree nor disagree, 3 = Agree, 4 = Strongly agree; Change in brushing technique: 1 = Much worse/Somewhat worse, 2 = Unchanged (the same), 3 = Somewhat better, 4 = Much better; Improved the way I brush: 1 = False, 2 = True

## Discussion

Understanding the impact of connected toothbrush use on user perceptions of brushing skills and behaviors and their everyday oral health (e.g., how clean teeth are, breath odor) is an important step in evaluating the potential benefits of these novel devices. We examined these perceptions in a single-arm study, where participants used an unconnected brush followed by use of a connected brush, and an RCT, where participants were randomized to use a connected or unconnected brush. The EOHAB, a novel PRO measure, was used to systematically quantify user perceptions during the studies.

The EOHAB measure provides a comprehensive method to examine patient perceptions regarding brushing behavior, oral health, and the use of a connected toothbrush system. Although we anticipated a unidimensional structure and a single score to emerge, 2 summary scores were derived from the EOHAB Item Set 1, which asks participants to reflect on their brushing skills, behaviors, and perceptions over the past 7 days. The items formed a total score and a specific “brushing experience” score that addresses brushing motivation, satisfaction, confidence, and amount of focus and attentiveness when brushing. Item Set 2 included impression of change items that asked directly about changes in brushing skills and behaviors since starting to use the study toothbrush. Rather than calculating an aggregate score for these items, they were examined individually. Item Set 3 includes items asking about the use of the smartphone app in the connected toothbrush system. Three scores were derived from this set: an Overall App Score, an App Utility Score with items assessing how useful the app was, and an App Usability Score that measured how easy it was to use the app. These item sets and scores provide a comprehensive assessment of the experience of using the connected brush system. The different item sets address different aspects of the user experience. Individual sets and scores can be selected for use in future trials according to the design of the study (e.g., the app scores may not be useful in a trial where some participants do not use the connected brush).

Item Set 1 Overall and Brushing Experience scores were very high at baseline in the single-arm study. This reflected a generally positive view regarding brushing behaviors and aspects of oral health prior to using the unconnected toothbrush and connected toothbrush. This limits the ability of the study toothbrushes to improve these user perspectives. In other words, a ceiling effect, where scores have limited room to increase during the study, may have impacted the scores from Item Set 1. This may, in part, underlie the failure to observe a significant impact of the connected toothbrush on these scores in the RCT. Ceiling effects may also limit variability in the scores, which could underlie the failure to observe meaningful relationships between the scores and the objective brushing and clinical metrics. However, this may also accurately reflect the absence of a relationship between brushing and oral health perceptions, as measured by the EOHAB, and connected brush use and clinical and objective measures of brushing and oral health.

Despite the high baseline scores, Overall and Brushing Experience scores incrementally improved following use of the unconnected electric brush and connected brush in the single-arm study; brushing and oral health perspectives were most positive after 1 week of connected brush use. However, both scores then leveled off and numerically decreased after 3 weeks of connected brush use, which may suggest a time-limited effect of the connected brush, user fatigue, or that the baseline ceiling effect limits the ability of the scale to show continued improvement over time.

The Item Set 2 impression of change items explicitly ask about changes in brushing skills and behaviors and were only administered following use of the study toothbrushes. Therefore, they were inherently immune to ceiling effects that may have influenced the Item Set 1 scores. Several of the items were improved following use of the connected brush in both the RCT and single-arm study. For example, in the single-arm study, approximately 95% of participants reported that the way that they brush had improved following 2 weeks of app use vs. approximately 40% after 1 week of electronic brush use alone. The impression of change items also exhibited stronger associations with the objective brushing variables than the scores from Item Set 1. By asking directly about change over time, these items may more accurately reflect changes in perspectives on brushing skills and behaviors. However, in the RCT, the effect of the connected brush on the impression of change items was diminished, which also suggests a potential time-limited effect on perceptions of brushing and oral health.

A separate item set was exclusively dedicated to understanding perspectives on the smartphone app that is used in the connected brush system. These items are unique to study contexts where participants use the smartphone app and, therefore, were not administered in the RCT where participants could be randomized to the unconnected brush group. In the observational study, both the App Utility and App Usability ratings were high (> 8.5 on 0–10 scale) after 1- and 3-weeks of connected brush use, although ratings decreased slightly in the later interval. This is also consistent with the pattern observed on the other EOHAB scores, where positive changes in perceptions seem to diminish over time. However, only the App Utility score was associated with the objective brushing variables. This is not surprising, as the App Utility score includes items about how useful the app is in improving brushing habits, which should be related to actual brushing behaviors. The App Usability score may be more likely to be related to initial adoption and continued use of the connected toothbrush (e.g., items about how easy it was to set up and use the app).

The studies had several limitations. Different EOHAB versions were used in the RCT and single-arm study, which limited the ability to compare how scores changed over time in both studies. A limited number of items were included in the RCT to decrease participant burden and as an initial pilot test of the measure. Participants in the single-arm study always used the connected brush after the non-connected brush which may have resulted in an order effect that differentially benefitted the connected brush. Additionally, the use of the connected brush was only evaluated for a maximum of 6 weeks. Future studies could include longer intervals to address how extended use of the connected toothbrush impacts perceptions on brushing and oral health. It is likely that a longer assessment interval would be required to fully understand the trajectory of changes in brushing and oral health perspectives following initiating use of a connected brush and to understand if any cognitive or behavioral changes are transient or more long lasting. In addition to extending the assessment interval, future studies examining the relationships with connected brushes and brushing metrics or clinical outcomes may also benefit from enrolling participants with a more heterogeneous mix of baseline EOHAB scores to avoid potential ceiling effects or response biases that could impact the ability to detect relationships with other variables. Including a more equal mix of male and female participants may also be useful. Although the diversity of the sample in the studies is an overall strength, the RCT was relatively less diverse than the single-arm study, which may have impacted the responses on the EOHAB.

### Conclusions

The use of a connected toothbrush improved several, but not all, perceptions of brushing skills and oral health in a diverse sample of participants, particularly as measured by items where respondents directly rate the changes that they have experienced over time. This is important, as some of these perceptions were related to brushing duration and coverage. However, they were unrelated to clinical outcomes in our study, which are ultimately the most important outcomes to impact. Our study suggests that the improvements in oral health and brushing perceptions may begin to level off or return to baseline levels after several weeks of connected brush use. Methods to enhance engagement with connected toothbrush systems over time may be useful to maintain any improvements in brushing skills and behaviors. The EOHAB provides a comprehensive measure of brushing-related perceptions and views on the connected toothbrush smartphone app. Researchers can select or modify EOHAB items to match specific study contexts and patient populations in future clinical trials with connected toothbrush systems. Additionally, incorporating items from the EOHAB into the connected brush system itself may eventually be a valuable method to assess the user experience in real-world clinical and consumer use settings.

## Data Availability

No datasets were generated or analysed during the current study.

## Abbreviations

BL1: Baseline Period 1
BL2: Baseline Period 2
CFA: Confirmatory Factor Analysis
CFI: Confirmatory Fit Index
EOHAB: Everyday Oral Health and Brushing
EFA: Exploratory Factor Analysis
LSGI: Löe-Silness Gingival Index
PRO: Patient-Reported Outcome
RCT: Randomized controlled trial
RM-NPI: Rustogi Modification of Navy Plaque Index
RMSEA: Root Mean Square Error of Approximation
SD: Standard deviation
TLI: Tucker-Lewis Index
WLSMV: Weighted least squares estimation with mean and variance correction
